# Differential Effect of Metabolic Health and Obesity on Incident Heart Failure: A Nationwide Population-Based Cohort Study

**DOI:** 10.3389/fendo.2021.625083

**Published:** 2021-02-25

**Authors:** Hwi Seung Kim, Jiwoo Lee, Yun Kyung Cho, Joong-Yeol Park, Woo Je Lee, Ye-Jee Kim, Chang Hee Jung

**Affiliations:** ^1^ Department of Internal Medicine, Asan Medical Center, University of Ulsan College of Medicine, Seoul, South Korea; ^2^ Asan Diabetes Center, Asan Medical Center, Seoul, South Korea; ^3^ Department of Internal Medicine, Hallym University Sacred Heart Hospital, Hallym University College of Medicine, Anyang, South Korea; ^4^ Department of Clinical Epidemiology and Biostatistics, Asan Medical Center, University of Ulsan College of Medicine, Seoul, South Korea

**Keywords:** obesity, metabolic syndrome, heart failure, metabolic health, metabolically healthy obese, risk factors

## Abstract

**Background:**

Metabolically healthy obese (MHO) individuals and their association with cardiometabolic diseases have remained controversial. We aimed to explore the risk of incident heart failure (HF) based on the baseline metabolic health and obesity status as well as their transition over 2 years.

**Methods:**

The Korean National Health Insurance Service-National Health Screening Cohort data of 514,886 participants were analyzed. Obesity was defined as BMI ≥25 kg/m^2^ according to the Korean Centers for Disease Control and Prevention. The metabolic health and obesity status were evaluated at baseline and after two years. Study participants were followed to either the date of newly diagnosed HF or the last follow-up visit, whichever occurred first.

**Results:**

The MHO group comprised 9.1% of the entire population and presented a better baseline metabolic profile than the metabolically unhealthy non-obese (MUNO) and metabolicavlly unhealthy obese (MUO) groups. During the median 71.3 months of follow-up, HF developed in 5,406 (1.5%) participants. The adjusted hazard ratios [HRs (95% CI)] of HF at baseline compared with the metabolically healthy non-obese (MHNO) group were 1.29 [1.20–1.39], 1.37 [1.22–1.53], and 1.63 [1.50–1.76] for MUNO, MHO, and MUO groups, respectively. With the stable MHNO group as reference, transition into metabolically unhealthy status (MUNO and MUO) increased the risk of HF, regardless of the baseline status. Subjects who were obese at both baseline and follow-up showed an increased risk of HF, regardless of their metabolic health status.

**Conclusions:**

Metabolic health and obesity status and their transition can predict the risk of incident HF. Losing metabolic health in baseline non-obese and obese individuals and remaining obese in baseline obese individuals showed a significantly increased risk of incident HF. Maintaining good metabolic health and a lean body may prevent the development of HF.

## Introduction

Obesity is a major global health issue, as the prevalence of obesity has constantly increased to a degree that almost one-third of the population worldwide is either overweight or obese (1). Obesity leads to numerous chronic diseases, such as type 2 diabetes, cardiovascular diseases (CVDs), cancer, musculoskeletal disorders, and psychological disorders (2); however, huge individual variability is observed in the risk of metabolic and clinical morbidity associated with obesity (3, 4). Among the obese population, the subgroup that does not exhibit cardiovascular and metabolic outcomes of obesity is classified as metabolically healthy obese (MHO) (5). MHO individuals are characterized by normal blood pressure (BP), favorable lipid profiles, and better insulin sensitivity despite their increased adiposity and can be exempted from increased risk of obesity-related disorders (6, 7); however, the prognostic value of MHO is debatable and it might depend on the health outcomes being tested (5, 8). Furthermore, MHO is not a fixed state, as metabolic health is a dynamic concept that can change over time. Accordingly, alteration of an individual’s metabolic health and obesity status over time should be considered while evaluating the risk of obesity-related disorders (4).

Among the various CVDs, heart failure (HF) has been an important public health concern worldwide due to its high morbidity and mortality (9). Obesity is a key determinant of cardiovascular health and is an independent risk factor for the development of HF (10, 11). Nevertheless, increased body mass index (BMI) causes left ventricular remodeling and neurohormonal alterations (12, 13). Moreover, higher BMI is frequently accompanied by numerous metabolic disorders characterized by hypertension, type 2 diabetes, and dyslipidemia, all of which are risk factors for HF (11, 14). Therefore, the importance of obesity *per se* as an independent contributor to HF, irrespective of obesity-induced metabolic disturbances, remains uncertain. Furthermore, being metabolically unhealthy, regardless of BMI, is generally associated with increased risk of CVD including HF, and normal weight does not necessarily indicate metabolic health (15–18). Therefore, how metabolic health and obesity distinctly affect the risk of HF development remains inconclusive.

Based on these backgrounds, we aimed to investigate the effect of metabolic health and obesity status on the risk of developing HF in a large national population-based cohort. Considering the dynamic nature of metabolic health and obesity, we also assessed the impact of transition in metabolic health and obesity status over two years on the risk of incident HF.

## Materials and Methods

### Data Source

The present study was based on the data provided by the Korean National Health Insurance Service (NHIS). The Korean NHIS requires all Korean citizens to register for national health care insurance. The use of health services, prescription or procedure records, and the 10th revision of the International Statistical Classification of Diseases and Related Health Problems (ICD-10) diagnosis codes is documented in the NHIS database. NHIS provides health screening examinations biennially for all those registered in the NHIS. The health examination includes anthropometry, comorbidities, family history, self-reported health behavior, and laboratory results. The NHIS-National Health Screening Cohort (NHIS-HEALS) 2002–2015 data was released in 2017, and ever since, it has been used as representative of the Korean population in research. This cohort comprises a random selection of 514,866 subjects, corresponding to about 10% of the entire population that underwent NHIS health exams between 2002 and 2003. This sample of individuals was followed up until death, emigration, or the end of the study period in 2015. The detailed structure and function of Korean NHIS-HEALS have been described elsewhere (19). Because this database was anonymized and de-identified, informed consent was waived. This study conforms to the ethical guidelines of the 1975 Declaration of Helsinki and was approved by the Asan Medical Center (Seoul, Korea) Institutional Review Board (IRB-No 2020-0852).

### Study Population

The present study examined the NHIS-HEALS data from January 1, 2009 to December 31, 2010, as the baseline data. This period was selected because laboratory results such as triglyceride (TG) and high-density lipoprotein cholesterol (HDL-C) were included from 2009 (19). The follow-up data were collected from January 1, 2011 to December 31, 2012, to analyze the effect of change in metabolic health and obesity. Of the initially selected 514,866 subjects—after excluding the deceased before the year 2012 (*n* = 33,358), those with a previous history of HF before 2012 (*n* = 20,063), those with missing measurements of BP, BMI, fasting plasma glucose (FPG), TG, or HDL-C (*n* = 105,130), and those with BMI >40 kg/m^2^ at baseline (*n* = 57)**—**eventually 356,258 subjects were included in the analysis.

### Definition of Metabolic Health and Obesity Status

Metabolic health was determined by the presence of risk factors defined according to the Adult Treatment Panel III (ATP III) criteria. The risk factors were as follows: (1) systolic BP ≥130 mmHg and/or diastolic BP ≥85 mmHg and/or use of antihypertensive medications; (2) TG ≥150 mg/dl and/or use of lipid-lowering medications; (3) FPG ≥100 mg/dl and/or use of antidiabetic medications; and (4) HDL-C <40 mg/dl in men and <50 mg/dl in women. A metabolically healthy status was defined as having none or one of the above-mentioned criteria. Obesity was defined as body mass index (BMI) ≥25 kg/m^2^, based on Asia–Pacific criteria, organized by the World Health Organization Western Pacific Region (20). Korean medical and governmental organizations including Korean Centers for Disease Control and Prevention have officially applied this definition of obesity (21). All subjects were divided into four groups based on the metabolic health and obesity criteria:

1. metabolically healthy non-obese (MHNO) group, defined as BMI <25 kg/m^2^ and none or one metabolic risk factor;
2. metabolically unhealthy non-obese (MUNO) group, defined as BMI <25 kg/m^2^ and two or more metabolic risk factors;
3. metabolically healthy obese (MHO) group, defined as BMI ≥25 kg/m^2^ and none or one metabolic risk factor; and
4. metabolically unhealthy obese (MUO) group, defined as BMI ≥25 kg/m^2^ and two or more metabolic risk factors.

### Study Outcome

The primary outcome of this study was incident HF during the study period. Newly diagnosed HF was defined as at least one new inpatient and/or outpatient care for HF, with the diagnostic code of I50 as a principal or subsidiary diagnosis (22, 23). The risk of incident HF was analyzed by baseline metabolic health and obesity status as well as by the transition of the status after a 2-year follow-up period.

### Definitions of Type 2 Diabetes, Hypertension, and Dyslipidemia

People who were prescribed antidiabetic drugs and those with ICD-10 codes E11 (non-insulin-dependent diabetes mellitus), E12 (malnutrition-related diabetes mellitus), E13 (other specified diabetes mellitus), and E14 (unspecified diabetes mellitus) were defined as having type 2 diabetes. The following eight classes of antidiabetic drugs were distributed by pharmacies in Korea during the study period: sulfonylureas, biguanides, *α*-glucosidase inhibitors, thiazolidinediones, meglitinides, glucagon-like peptide-1 receptor agonists, dipeptidyl peptidase-4 inhibitors, and insulin.

People who were prescribed antihypertensive medications and those with ICD-10 codes I10 [essential (primary) hypertension], I11 (hypertensive heart disease), I12 [hypertensive chronic kidney disease (CKD)], I13 (hypertensive heart and CKD), and I15 (secondary hypertension) were defined as having hypertension. The following five classes of antihypertensive medications were distributed by pharmacies in Korea during the study period: angiotensin receptor blockers, angiotensin-converting enzyme inhibitors, beta-blockers, calcium-channel blockers, and diuretics. People who were prescribed lipid-lowering medications and those with ICD-10 code E78 (disorders of lipoprotein metabolism and other lipidemias) were defined as having dyslipidemia. Lipid-lowering medications distributed by pharmacies in Korea during the study period included statins, ezetimibe, and fibrates.

### Covariates

Covariates included age, sex, income, health-related behaviors, such as drinking (none, mild, moderate, or heavy drinking), smoking (non-, ex-, or current smoker), and physical activities (0, 1–2, 3–4, or ≥5 times per week), total cholesterol (TC), estimated glomerular filtration rate (eGFR), and associated medical history and comorbidities, such as atrial fibrillation (AF), ischemic heart disease (IHD), and chronic obstructive pulmonary disease (COPD). Heavy drinkers were defined as individuals consuming ≥seven drinks on one occasion and drinking >5 days per week, whereas mild and moderate drinkers were those consuming <seven drinks in one day and drinking 1–2 or 3–4 days per week, respectively (24, 25). The definitions of diseases mentioned are listed in **Supplement Table 1**.

The eGFR was calculated using the Chronic Kidney Disease Epidemiology Collaboration equation as follows (26): GFR = 141 × min (Scr/*κ*, 1)*^α^* × max (Scr/*κ*, 1)^−1.209^ × 0.993^Age^ × 1.018 (if female), where Scr is serum creatinine, *κ* is 0.7 for females and 0.9 for males, *α* is −0.329 for females and −0.411 for males, and min and max indicate the minimum and maximum of Scr/*κ* or 1, respectively.

### Statistical Analysis

Continuous variables were presented as means ± standard deviation (SD), and categorical variables as percentages (%). To compare the baseline characteristics of the study participants based on their metabolic health and obesity status, analysis of variance (ANOVA) and Scheffe’s test for *post-hoc* analysis or chi-square test was employed. Multiple imputation procedure was performed to impute the missing variables. The hazard ratio (HR) and 95% confidence interval (CI) for incident HF during the follow-up period for each group were determined by performing Cox proportional hazards analyses. Model 1 was adjusted for age, sex, and income; model 2 was further adjusted for smoking, alcohol drinking, and physical activities; model 3 was adjusted for TC, eGFR (eGFR <60 ml/min/1.73 m^2^ or ≥60 ml/min/1.73 m^2^), AF, IHD, and COPD as well as for the variables included in model 2.

Initially, the risk of incident HF was assessed in terms of the baseline metabolic health and obesity status, with the MHNO group being considered as the reference group. Next, the transition of metabolic health and obesity status over 2 years, with the stable MHNO group as the reference group, was further analyzed. Statistical analyses were performed using the SAS Enterprise Guide software (version 7.1, SAS Institute, Inc., Cary, NC).

## Results

### Baseline Characteristics of the Study Population

The baseline characteristics of the study participants are presented in **Table 1**. The MHNO, MUNO, MHO, and MUO groups each comprised 29.8% (*n* = 106,289), 34.4% (*n* = 122,455), 9.1% (*n* = 32,547), and 26.7% (*n* = 94,967), respectively, of the entire study population. As expected, the baseline metabolic parameters showed significant differences between groups. Compared with the MHNO group, the MHO group revealed worse overall metabolic parameters with elevated FPG, TG, low-density lipoprotein cholesterol (LDL-C), and TC levels and reduced HDL-C level. Compared with the MUNO and MUO groups, the MHO group presented a favorable metabolic profile, including lower FPG and TG levels and higher HDL-C levels. Some unusual findings were observed. The mean TC levels in the MHO group were marginally above normal, and the systolic and diastolic BP levels were significantly higher in the MHO group compared with the MHNO group.

**Table 1:** Characteristics of study participants according to baseline metabolic health and obesity status.

Obesity category	Non-obese (BMI < 25 kg/m^2^)		Obese (BMI ≥ 25 kg/m^2^)		*P* value
Risk factor	0–1 risk factor	≥2 risk factors	0–1 risk factor	≥2 risk factors	
	MHNO	MUNO	MHO	MUO	
*n* (%)	106,289 (29.8)	122,455 (34.4)	32,547 (9.1)	94,967 (26.7)	
Sex (% male)	48.8	56.0	50.7	58.6	<0.001
Age (years)	57.5 ± 5.4^*^	60.0 ± 8.9	57.5 ± 7.8^*^	59.0 ± 8.3	<0.001
BMI (kg/m^2^)	22.1 ± 1.9	22.7 ± 1.7	26.8 ± 1.6	27.2 ± 1.9	<0.001
WC (cm)	77.1 ± 6.7	80.4 ± 6.4	86.9 ± 6.4	89.4 ± 6.5	<0.001
SBP (mmHg)	119.0 ± 13.9	129.7 ± 14.5	123.4 ± 14.1	132.2 ± 14.3	<0.001
DBP (mmHg)	73.9 ± 9.3	80.4 ± 9.4	76.4 ± 9.5	82.0 ± 9.6	<0.001
Current Smoker (%)	15.8	19.9	13.1	17.8	<0.001
Drinking (%)					<0.001
None	60.5	55.7	58.1	52.8	
Mild	18.6	16.5	17.8	16.2	
Moderate	4.0	4.8	4.0	4.3	
Heavy	14.7	20.6	17.6	24.3	
Physical activity (%)					<0.001
None	26.5	29.1	27.2	29.0	
1–2 times/week	22.3	22.0	22.8	23.6	
3–4 times/week	21.6	20.7	21.2	20.9	
≥5 times/week	27.9	26.1	26.8	24.4	
Medical history (%)					
Type 2 diabetes	2.1	15.3	2.5	18.1	<0.001
Hypertension	13.0	43.9	22.1	54.8	<0.001
Dyslipidemia	4.1	29.7	4.4	35.0	<0.001
Atrial fibrillation	0.6	1.1	0.7	1.1	<0.001
IHD	5.1	12.1	6.3	14.5	<0.001
COPD	0.7	0.9	0.5	0.7	<0.001
FPG (mg/dl)	91.6 ± 12.8	108.7 ± 29.7	92.3 ± 12.1	110.7 ± 29.7	<0.001
TG (mg/dl)	97.7 ± 46.3	166.9 ± 102.3	109.1 ± 49.4	189.2 ± 111.6	<0.001
LDL-C (mg/dl)	120.0 ± 33.8	121.4 ± 40.8	125.9 ± 4.2	122.8 ± 42.0	<0.001
HDL-C (mg/dl)	59.5 ± 25.9	51.5 ± 26.6	56.8 ± 24.6	49.2 ± 23.4	<0.001
TC (mg/dl)	198.3 ± 33.2	204.1 ± 39.9^*^	203.7 ± 33.8^*^	207.2 ± 39.9	<0.001
eGFR (ml/min/1.73m^2^)	82.6 (18.3)	78.5 (19.6)	81.3 (18.0)	77.7 (19.5)	<0.001
eGFR <60 ml/min/1.73m^2^ (%)	6.7	11.2	7.2	11.9	<0.001

Results reported as means ± SD (standard deviation), unless otherwise indicated.

All variables were statistically different among the four groups, unless otherwise indicated. ^*^No statistical difference was observed.

BMI, body mass index; COPD, chronic obstructive pulmonary disease; DBP, diastolic blood pressure; eGFR, estimated glomerular filtration rate; FPG, fasting plasma glucose; HDL-C, high-density lipoprotein-cholesterol; IHD, ischemic heart disease; LDL-C, low-density lipoprotein-cholesterol; MHNO, metabolically healthy non-obese group; MHO, metabolically healthy obese group; MUNO, metabolically unhealthy non-obese group; MUO, metabolically unhealthy obese group; SBP, systolic blood pressure; TC, total cholesterol; TG, triglyceride; WC, waist circumference.

### Incident HF According to Baseline Metabolic Health and Obesity Status

**Figure 1:**
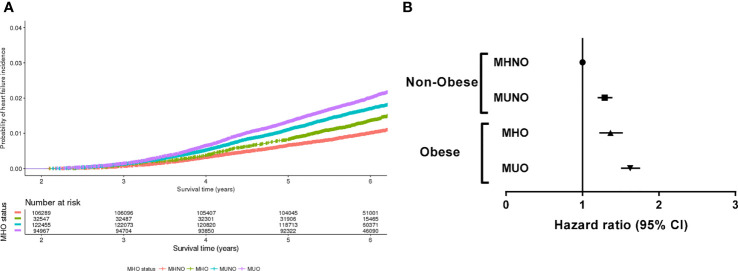
Risk of incident heart failure according to the metabolic health and obesity status at baseline. **(A)** Heart failure-free survival curves of four groups divided by the baseline metabolic health and obesity status. **(B)** Hazard ratios for the development of heart failure according to the baseline metabolic health and obesity status.

During the follow-up period (median, 71.3 months), 5,406 of the 356,258 individuals (1.5%) developed incident HF. The crude incidence rate of HF was 1.0% (1,053/106,289) in the MHNO group, 1.7% (2,038/122,455) in the MUNO group, 1.3% (432/32,547) in the MHO group and 2.0% (1,883/94,967) in the MUO group. **Figure 1A** presents the HF-free survival curves for the four groups defined by the baseline metabolic health and obesity state.

**Table 2** and **Figure 1B** demonstrate the HRs for incident HF according to the baseline metabolic health and obesity status. Compared with the MHNO group, the risk of HF significantly increased in the MUNO, MHO, and MUO groups after complete adjustment (**Table 2**, model 3). The MUNO, MHO, and MUO groups presented HRs of 1.29 (95% CI, 1.20–1.39), 1.37 (95% CI, 1.22–1.53), and 1.63 (95% CI, 1.50–1.76), respectively.

**Table 2:** Risk of incident HF according to baseline metabolic health and obesity status defined by BMI criteria.

Obesity category	Non-obese (BMI < 25 kg/m^2^)		Obese (BMI ≥ 25 kg/m^2^)	
Risk factor	0–1 risk factor	≥2 risk factors	0–1 risk factor	≥2 risk factors
	MHNO	MUNO	MHO	MUO
*n* (% of total)	106,289 (29.8)	122,455 (34.4)	32,547 (9.1)	94,967 (26.7)
HF event incidence				
Number of events (%)	1,053 (1.0)	2,038 (1.7)	432 (1.3)	1,883 (2.0)
Incidence/1000 person-years (95% CI)	1.68 (1.58–1.79)	2.82 (2.70–2.95)	2.26 (2.05–2.48)	3.37 (3.22–3.52)
Crude HR (95% CI)	1.00 (ref)	1.67 (1.55–1.80)	1.34 (1.20–1.50)	2.00 (1.85–2.15)
Multivariable-adjusted HR (95% CI)				
Model 1^a^	1.00 (ref)	1.41 (1.31–1.52)	1.38 (1.23–1.54)	1.83 (1.70–1.97)
Model 2^b^	1.00 (ref)	1.41 (1.31–1.51)	1.38 (1.24–1.55)	1.83 (1.69–1.97)
Model 3^c^	1.00 (ref)	1.29 (1.20–1.39)	1.37 (1.22–1.53)	1.63 (1.50–1.76)

AF, atrial fibrillation; BMI, body mass index; CI, confidence interval; COPD, chronic obstructive pulmonary disease; eGFR, estimated glomerular filtration rate; HF, heart failure; HR, hazard ratio; IHD, ischemic heart disease; MHNO, metabolically healthy non-obese group; MHO, metabolically healthy obese group; MUNO, metabolically unhealthy non-obese group; MUO, metabolically unhealthy obese group.

a Adjusted for age, sex, and income.

b Adjusted for age, sex, income, smoking, alcohol drinking, and physical activities.

c Adjusted for age, sex, income, smoking, alcohol drinking, physical activities, total cholesterol, eGFR(<60mL/min/1.73m^2^), AF, IHD, and COPD.

### Incident HF According to the Transition of Metabolic Health and Obesity Status

To further analyze the differential effect of obesity and metabolic health on the risk of incident HF, we separately calculated the HRs for incident HF in the baseline non-obese (**Table 3**) and obese (**Table 4**) groups by considering their transition over the 2 years. As listed in **Table 3**, in the baseline MHNO group, the transition to the metabolically unhealthy, *i.e.*, MUNO and MUO, increased the risk of incident HF with HR values of 1.33 (95% CI, 1.15–1.54) and 1.67 (95% CI, 1.21–2.32), respectively after full adjustment (**Table 3**, model 3). Similarly, in the baseline MUNO group, maintenance of metabolically unhealthy status (*i.e.*, stable MUNO and MUNO to MUO) significantly increased the risk of incident HF (HR 1.47, 95% CI, 1.33–1.64 for stable MUNO and HR 1.68, and 95% CI, 1.40–2.01 for MUNO to MUO, respectively), compared with that for stable MHNO group (**Table 3**, model 3). On the other hand, becoming obese without deterioration of metabolic risk factors, *i.e.*, transitioning from MHNO to MHO, did not alter the risk of incident HF (HR 0.71; 95% CI: 0.45–1.11), implying that the obesity aspect may not raise the risk of incident HF in metabolically healthy status.

**Table 3:** Risk of incident HF in participants according to baseline and 2-year metabolic health and obesity status among non-obese subjects at baseline.

Baseline	Follow-up	*n* (%)	HF event (*n*, %)	Incidence rate	Crude HR	Multivariable HR		
						Model 1^a^	Model 2^b^	Model 3^c^
**MHNO**	**MHNO**	63,934 (69.2)	519 (0.8)	1.38 (1.26–1.50)	1.00 (ref)	1.00 (ref)	1.00 (ref)	1.00 (ref)
	**MUNO**	22,348 (24.2)	298 (1.3)	2.26 (2.01–2.53)	1.64 (1.42–1.89)	1.34 (1.16–1.55)	1.34 (1.16–1.55)	1.33 (1.15–1.54)
	**MHO**	3,599 (3.9)	20 (0.6)	0.94 (0.57–1.45)	0.68 (0.44–1.07)	0.72 (0.46–1.13)	0.72 (0.46–1.13)	0.71 (0.45–1.11)
	**MUO**	2,524 (2.7)	39 (1.6)	2.61 (1.86–3.57)	1.90 (1.37–2.62)	1.72 (1.25–2.39)	1.72 (1.24–2.38)	1.67 (1.21–2.32)
**P-value**					<.001	<.001	<.001	0.001
**MUNO**	**MHNO**	27,642 (26.2)	289 (1.1)	1.76 (1.57–1.98)	1.27 (1.10–1.47)	1.14 (0.99–1.32)	1.14 (0.99–1.32)	1.10 (0.96–1.28)
	**MUNO**	68,008 (64.3)	1,179 (1.7)	2.93 (2.76–3.10)	2.12 (1.91–2.35)	1.66 (1.50–1.85)	1.66 (1.50–1.85)	1.47 (1.33–1.64)
	**MHO**	1,932 (1.8)	21 (1.1)	1.83 (1.13–2.80)	1.32 (0.85–2.04)	1.22 (1.79–1.89)	1.23 (0.79–1.90)	1.20 (0.78–1.86)
	**MUO**	8,119 (7.7)	154 (1.9)	3.21 (2.73–3.76)	2.33 (1.95–2.79)	1.92 (1.60–2.30)	1.92 (1.60–2.30)	1.68 (1.40–2.01)
**P-value**					<.001	<.001	<.001	<.001

Incidence rate and HR were presented as “/1,000 person-years (95% CI)” and “HR (95% CI)”, respectively. P-value was calculated by test for trend among the subset of baseline MHO status, in order of follow-up MHO status and stable MHNO group as a reference.

MHNO, metabolically healthy non-obese; MHO, metabolically healthy obese; MUNO, metabolically unhealthy non-obese; MUO, metabolically unhealthy obese.

a Adjusted for age, sex, and income.

b Adjusted for age, sex, income, smoking, alcohol drinking, and physical activities.

c Adjusted for age, sex, income, smoking, alcohol drinking, physical activities, total cholesterol, eGFR(<60mL/min/1.73m^2^), AF, IHD, and COPD.

**Table 4:** Risk of incident HF in participants according to baseline and 2-year metabolic health and obesity status among obese subjects. at baseline.

Baseline	Follow-up	*n* (%)	HF event (*n*, %)	Incidence rate	Crude HR	Multivariable HR		
						Model 1^a^	Model 2^b^	Model 3^c^
**MHNO**	**MHNO**	63,934 (69.2)	519 (0.8)	1.38 (1.26–1.50)	1.00 (ref)	1.00 (ref)	1.00 (ref)	1.00 (ref)
**MHO**	**MHNO**	3,392 (12.0)	26 (0.8)	1.30 (0.85–1.90)	0.94 (0.63–1.39)	0.89 (0.60–1.31)	0.88 (0.59–1.31)	0.86 (0.58–1.28)
	**MUNO**	1,605 (5.7)	24 (1.5)	2.52 (1.62–3.75)	1.82 (1.21–2.74)	1.43 (0.95–2.15)	1.42 (0.94–2.14)	1.39 (0.92–2.09)
	**MHO**	13,316 (47.1)	165 (1.2)	2.10 (1.80–2.45)	1.53 (1.29–1.83)	1.64 (1.37–1.95)	1.62 (1.36–1.93)	1.61 (1.35–1.92)
	**MUO**	9,940 (35.2)	157 (1.6)	2.68 (2.27–3.13)	1.94 (1.63–2.32)	1.79 (1.50–2.15)	1.78 (1.49–2.13)	1.75 (1.46–2.09)
**P-value**					<.001	<.001	<.001	<.001
**MUO**	**MHNO**	3,022 (3.7)	26 (0.9)	1.45 (0.95–2.12)	1.04 (0.70–1.55)	0.95 (0.64–1.40)	0.94 (0.63–1.39)	0.91 (0.61–1.35)
	**MUNO**	9,503 (11.5)	202 (2.1)	3.58 (3.10–4.11)	2.57 (2.18–3.02)	1.99 (1.69–2.34)	1.97 (1.67–2.32)	1.76 (1.49–2.07)
	**MHO**	11,447 (13.9)	163 (1.4)	2.41 (2.05–2.81)	1.74 (1.46–2.08)	1.72 (1.44–2.05)	1.71 (1.43–2.03)	1.65 (1.38–1.97)
	**MUO**	58,403 (70.9)	1,232 (2.1)	3.58 (3.38–3.78)	2.60 (2.35–2.88)	2.30 (2.07–2.55)	2.28 (2.05–2.53)	2.01 (1.80–2.23)
**P-value**					<.001	<.001	<.001	<.001

Incidence rate and HR were presented as “/1,000 person**–**years (95% CI)” and “HR (95% CI)”, respectively. P-value was calculated by test for trend among the subset of baseline MHO status, in order of follow-up MHO status and stable MHNO group as a reference.

MHNO, metabolically healthy non-obese; MHO, metabolically healthy obese; MUNO, metabolically unhealthy non-obese; MUO, metabolically unhealthy obese.

a Adjusted for age, sex, and income.

b Adjusted for age, sex, income, smoking, alcohol drinking, and physical activities.

c Adjusted for age, sex, income, smoking, alcohol drinking, physical activities, total cholesterol, eGFR(<60 ml/min/1.73m^2^), AF, IHD, and COPD.

**Figure 2:**
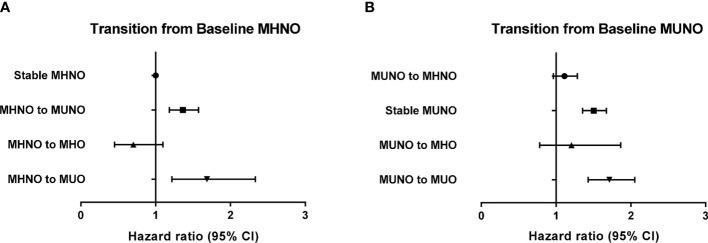
Risk of incident heart failure according to the transition of metabolic health and obesity status among non-obese individuals at baseline in reference to the stable MHNO group. **(A)** Transition from baseline MHNO, **(B)** transition from baseline MUNO.

**Figure 3:**
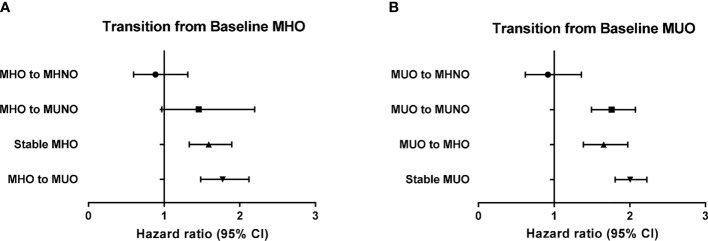
Risk of incident heart failure according to the transition of metabolic health and obesity status among obese individuals at baseline in reference to the stable MHNO group. **(A)** Transition from baseline MHO, **(B)** transition from baseline MUO.

**Table 4** shows the risk of incident HF in the baseline obese population. Among the subjects with MHO at baseline, 12.0% (*n* = 3392) achieved weight loss while maintaining their metabolic health (MHO to MHNO), which conferred no significant increase in the risk of incident HF (HR 0.86, 95% CI: 0.58–1.28), compared with that in the stable MHNO group. However, both remaining at the stable MHO condition and transitioning to unhealthy status irrespective of their obesity status (*i.e.*, MHO to MUNO and MHO to MUO group) revealed an increased risk of incident HF, although the HR of MHO to MUNO group was statistically marginal (HR 1.39, 95% CI, 0.92–2.09, *p*-value = 0.071, **Table 4**). Similarly, among the baseline MUO participants, who maintained their obesity status regardless of their metabolic health status (MUO to MHO and stable MUO) and retained their unhealthy status despite their body weight reduction (MUO to MUNO), there was a significantly increased risk of incident HF (**Table 4**). **Figures 2** and **3** summarize the associations of metabolic health and obesity with incident HF, thereby focusing on the transitions in metabolic health and obesity status.

## Discussion

In this large, population-based study, we demonstrated that the risk of incident HF increased in the metabolically unhealthy obese and non-obese subjects, as well as in metabolically healthy obese subjects (MUNO, MUO, and MHO), compared to that observed in the metabolically healthy non-obese population (MHNO). Longitudinal assessment of the participants’ metabolic health and obesity status further indicated that among the non-obese population, metabolically unhealthy state at follow-up regardless of weight gain significantly increased the risk of incident HF (**Table 3** and **Figure 2**), thereby highlighting the crucial role of metabolic health as a contributing factor to incident HF. In contrast, among those subjects with obesity at baseline, either losing metabolic health (MHO becoming MUO or MHO becoming MUNO) or maintaining healthy obesity (stable MHO) was associated with increased risk of HF in comparison with the stable MHNO as the reference group (**Table 4** and **Figure 3**). Interestingly, the baseline MUO group was still associated with increased risk of HF, compared with the stable MHNO group as reference (**Table 4** and **Figure 3**), even after weight loss (MUO becoming MUNO) or restoring metabolic health (MUO becoming MHO), thus implicating the importance of obesity *per se* as a risk factor for incident HF. The stable MUO group showed the highest risk of incident HF (HR 2.01, 95% CI 1.80–2.23) (**Table 4** and **Figure 3**).

Obesity is a well-established risk factor for CVD, and it aggravates other CVD risk factors, such as insulin resistance, BP, dyslipidemia, and systemic inflammation (27). In general, obesity combined with other risk factors can alter the cardiovascular physiology and function, thereby resulting in the development of CVD, including hypertension, IHD, HF, peripheral artery disease, and AF (28). Obesity as an independent risk factor of HF was examined in the previous analysis of 5,881 participants in the Framingham Heart Study (11). One unit increase in BMI resulted in 5 and 7% increased risk of incident HF, each for men and women, respectively, even after adjustment of other known risk factors of HF (11). A similar pattern was observed in the Physicians’ Health Study, which reported an 11% increase in HF risk for one unit rise of BMI (29); however, a population of obesity without other CVD risk factors is yet to be investigated.

As the concept of obesity without cardiometabolic risk factors emerged, MHO and its association with the risk of obesity-related diseases have been questioned. Previous studies reported that MHO subjects, compared with MUO subjects, did not exhibit an increased risk of CVD (30, 31); however, only a few studies were conducted on the relationship between MHO and HF. In the Norwegian population-based cohort of 61,299 participants, the MHO subgroup was associated with increased risk of HF, but simultaneously it was not associated with increased risk of acute myocardial infarction, compared with the non-obese, metabolically healthy (MHNO) subgroup (32). The risk of HF increased with elevated BMI and long-lasting obesity, regardless of metabolic health (32). In another study, MHO individuals presented greater desynchrony and early diastolic dysfunction, compared with healthy individuals, suggesting that obesity, regardless of metabolic health status, is associated with cardiovascular risk (33). In contrast, the research performed in Greece demonstrated that the risk of HF increased in patients with metabolic syndrome, regardless of BMI (34). Although the previous studies claimed that either obesity or metabolic health was independently associated with increased risk of HF, how differently obesity and metabolic health affect the risk of incident HF remains unclear. To further characterize their possible differential effect on the development of HF, we performed a separate analysis based on the presence of obesity in our present study.

An individual’s metabolic health and obesity can change over time, and this dynamic nature of metabolic health and obesity status increases the risk of CVD (25, 35, 36). Previous studies reporting the transition in metabolic health and obesity in relation to the risk of HF are limited. One previous study followed patients with metabolic syndrome for 6 years and revealed that they were at 2.3, 2.6, and 2.1 (normal weight, overweight, and obese, respectively) times higher risk of developing HF than those without metabolic syndrome (34). Another recent longitudinal study revealed that the hospitalization for HF was increased in obese subjects who transitioned from metabolically healthy to unhealthy status (37). Among individuals without metabolic syndrome, obese individuals were at a higher risk of hospitalization for HF than non-obese individuals (37). The two previous studies classified patients according to their metabolic syndrome, which indicates meeting three or more of the five criteria, including waist circumference, BP, fasting TG level, fasting HDL-C, and FPG levels (38). In the present study, metabolic health was defined as having none or one of the five criteria, and the difference in the number of metabolic risk factors while sorting the participants may produce varying results. Nevertheless, our analysis on the transition of metabolic health and obesity status in the obese population also revealed an increased risk of HF in the subgroups that maintained obesity and/or acquired metabolic risk factors.

Among obese patients at baseline, reduced body weight without improvement in their metabolic health at follow-up did not decrease the risk of HF (**Table 4** and **Figure 3**). While the HR of developing HF among the MHO to MHNO group was 0.86 (95% HR 0.58–1.28), the incidence of HF among the MUO to MUNO and MUO to MUO groups was both 2.1%, and the HR of incident HF was 1.76 and 2.01, respectively (**Table 4** and **Figure 3**). Our result is contradictory to that of a recent study, dealing with the incidence of hospitalization for HF among metabolically healthy and obese individuals, which indicated that those who managed to lose weight and acquired the metabolically healthy and non-obese status were associated with a decreased risk of hospitalization for HF (37); however, as aforementioned, the criteria for metabolic health differed between the two studies. Moreover, the endpoint was set up differently, hospitalization for HF in the previous study and inpatient and/or outpatient care for HF in the present study.

In the present study, few individuals exhibited changes in obesity status over time. Overall, the baseline non-obese population is likely to remain non-obese; only 6.6% of the individuals in the MHNO group and 9.5% of the individuals in the MUNO group gained weight and became obese. Similarly, baseline obese subjects exhibited the tendency to stay obese, with merely 17.7 and 15.2% of the individuals in the MHO and MUO groups managed to lose weight. In particular, in the baseline MUO population, the HRs of incident HF were increased even in the subgroups, which showed improved metabolic health and became MHO or managed to reduce weight and became MUNO (**Table 4** and **Figure 3**). The obese population in Asia is characterized by visceral obesity, and visceral fat is known to promote systemic inflammation and induce metabolic derangements (39). Visceral fat is associated with left ventricular concentricity and remodeling, both of which are considered harbingers of HF (40, 41). Once visceral obesity initiates the inflammatory process, it may not be reversed by improving either metabolic health or obesity according to our results. Both metabolic health and normal weight may be able to reverse the pathological process.

Various mechanisms have been hypothesized to explain the association between obesity and HF. Adipose tissue deposition increases the circulating blood volume, thus requiring more left ventricular stroke volume and cardiac output (42). This results in enlargement and hypertrophy of the left ventricle, finally leading to left ventricular failure (42). During this process, hypertension can develop and contribute to HF development. Moreover, sleep apnea and hypoventilation due to obesity cause chronic hypoxia, resulting in pulmonary hypertension and right ventricular failure (43). Furthermore, neurohormonal alterations, involving aldosterone, leptin, and neprilysin, activate the sympathetic nervous and renin-angiotensin-aldosterone systems and induce fluid retention and cardiac remodeling (13, 44–46). Exacerbation of inflammation by acquiring insulin resistance and metabolic disturbances may also contribute to the initiation of HF (47). When obese participants remain obese and acquire cardio-metabolic risk factors, the pathophysiological mechanisms may be stimulated, thereby accelerating the process of HF development. The distinct process among MHO subjects remains unclear; however, MHO and MUO certainly have discrepancies in HF incidence, thereby predicting may be useful to prevent HF.

The concept of the “obesity paradox” indicates that individuals with high BMI have increased incidence of HF, but simultaneously present improved HF outcome, compared with those with low BMI (48). A previous study supported this “obesity paradox” by reporting higher CVD incidence and lower all-cause mortality in the Korean population (25). Our study examined only the development of incident HF as the outcome, so whether the “obesity paradox” applies to this subpopulation needs further assessment of the follow-up data.

This study had several limitations. First, only Koreans were included in the study; therefore, the results may not be generalized to other ethnic groups. Second, the follow-up duration is relatively short; hence, the number of HF events may be underestimated. Third, this study may have selection bias, as the age and sex of the present study population differ from those of the general Korean population. Fourth, due to the retrospective setting, despite adjustment for possible relevant confounders, the results of this study may have been influenced by such factors.

These limitations are complemented by the strengths of this study. First, we used large-scale, national population-based data. Second, the data analyzed in this study were longitudinal; hence, we could evaluate the evolution of metabolic health and obesity status as well as its relationship with the development of HF over time. Third, we adjusted for various confounding factors that might have affected the development of HF, including coexisting conditions as AF, IHD, and COPD.

In conclusion, an individual’s metabolic health and obesity as well as the transition of metabolic health and obesity status can predict the risk of incident HF. In non-obese individuals, losing metabolic health was the principal determinant for developing incident HF, whereas in obese individuals, either losing metabolic health or maintaining the obesity status was significantly associated with increased risk of HF. Therefore, efforts to concurrently preserve metabolic health and lose weight can be helpful in reducing the risk of developing HF.

## Data Availability Statement

The raw data supporting the conclusions of this article will be made available by the authors, without undue reservation.

## Ethics Statement

The studies involving human participants were reviewed and approved by Asan Medical Center Institutional Review Board (IRB-No 2020-0852). Written informed consent for participation was not required for this study in accordance with the national legislation and the institutional requirements.

## Author Contributions

CJ conceptualized this study. HK and CJ designed the study. HK, JL, and YC collected data. HK and Y-JK conducted the analysis. J-YP, WL, HK, Y-JK, and CJ interpreted the results. HK wrote the initial draft of the manuscript. All authors contributed to the article and approved the submitted version.

## Funding

This work was supported by a grant (2014-583, and 2015-583) from the Asan Institute of Life Sciences, Republic of Korea and by a grant (CJ, 2020F-2) from the Korean Diabetes Association. These funding sources had no roles in the design of the study, the collection, analysis, and interpretation of data, the writing of the article, or the decision to submit the article for publication.

## Conflict of Interest

The authors declare that the research was conducted in the absence of any commercial or financial relationships that could be construed as a potential conflict of interest.
