# Supplementary material for: Differential Effect of Metabolic Health and Obesity on Incident Heart Failure: A Nationwide Population-Based Cohort Study
Source: Front Endocrinol (Lausanne). 2021 Feb 25;12:625083. doi: 10.3389/fendo.2021.625083 (PMC7947792; doi:10.3389/fendo.2021.625083)
Supplement: Supplementary file 1 [file Table_1.docx]

**Supplemental Material**

**Supplement Table 1. Definitions of heart failure and comorbidities**

|  | ICD-10-CM codes and definitions | Diagnostic definition |
| --- | --- | --- |
| Heart failure | I50 | Admission or outpatient department ≥1 |
| Type 2 Diabetes mellitus | E11-14 | Admission or outpatient department ≥1 and anti-diabetic medication (sulfonylureas, biguanides, α-glucosidase inhibitors, thiazolidinediones, meglitinide, glucagon-like peptide-1 receptor agonists, dipeptidyl peptidase-4 inhibitors, and insulin) |
| Hypertension | I10-15 | Admission or outpatient department ≥1 and anti-hypertensive medication (angiotensin receptor blockers, angiotensin-converting enzyme inhibitors, beta blockers, calcium-channel blockers, and diuretics) |
| Dyslipidemia | E78 | Admission or outpatient department ≥1 and anti-dyslipidemia medication (included statins, ezetimibe, and fibrates) |
| Atrial fibrillation (AF) | I48.0–4, I48.9 | Admission or outpatient department ≥1 |
| Ischemic heart disease (IHD) | I20–25 | Admission or outpatient department ≥1 |
| Chronic obstructive pulmonary diseases (COPD) | J41–44 | Admission ≥1 |

The diagnostic code is based on ICD-10-CM used in the NHIS database, and the measurement is from the routine health checkup provided by the NHIS. The claims were searched in the NHIS database.

Abbreviation: ICD-10-CM =International Classification of Disease, Tenth Revision, Clinical Modification; NHIS =National Health Insurance Service
